# The non-primate hepacivirus 5′ untranslated region possesses internal ribosomal entry site activity

**DOI:** 10.1099/vir.0.055764-0

**Published:** 2013-12

**Authors:** Hazel Stewart, Cheryl Walter, Dale Jones, Sinead Lyons, Peter Simmonds, Mark Harris

**Affiliations:** 1 School of Molecular and Cellular Biology, Faculty of Biological Sciences and Astbury Centre for Structural Molecular Biology, University of Leeds, Leeds LS2 9JT, UK; 2 Infection and Immunity Division, The Roslin Institute, University of Edinburgh, Easter Bush, Edinburgh EH25 9RG, UK

## Abstract

The 5′ untranslated region (5′UTR) of the recently described non-primate hepacivirus (NPHV) contains a region with sequence homology to the internal ribosomal entry site (IRES) of hepatitis C virus (HCV) and GB virus B (GBV-B). Here, we demonstrated internal translation initiation by the NPHV 5′UTR in a bicistronic vector. An RNA stem–loop upstream of the NPHV IRES was structurally distinct from corresponding regions in HCV and GBV-B, and was not required for IRES function. Insertion of the NPHV stem–loop into the corresponding region of the HCV 5′UTR within the HCV subgenomic replicon significantly impaired RNA replication, indicating that long-range interactions between the 5′UTR and *cis*-acting downstream elements within the NPHV genome are not interchangeable with those of HCV. Despite similarities in IRES structure and function between hepaciviruses, replication elements in the NPHV 5′UTR appear functionally distinct from those of HCV.

Hepatitis C virus (HCV) infects approximately 170 million people worldwide and is the leading cause of chronic liver disease (Shepard *et al.*, 2005). There is an urgent need for relevant small-animal models and cell-culture systems to allow investigation into the virus–host interactions of this pathogen and the development of novel therapeutics. Non-primate hepacivirus (NPHV) is a closely related member of the genus *Hepacivirus* recently isolated from domestic dogs and horses, which may represent a potential HCV model (Burbelo *et al.*, 2012; Kapoor *et al.*, 2011). Although the natural host(s) of NPHV are not definitively established, only horses show evidence for widespread infection by serology and viraemia screening (Burbelo *et al.*, 2012; Lyons *et al.*, 2012). Horses are hosts to a wide range of flaviviruses, including the recently described Theiler’s disease-associated virus that is associated with acute hepatitis on primary infection (Chandriani *et al.*, 2013). However, the majority of infections with this virus are non-persistent and its genome organization and phylogenetic relationships place it within the genus *Pegivirus* of the family *Flaviviridae*.

Phylogenetically, NPHV therefore represents the closest known relative to HCV, although very recently a wide range of additional, highly divergent hepaciviruses and pegiviruses have been reported independently in multiple bat and rodent species (Kapoor *et al.*, 2013; Quan *et al.*, 2013). Given the widespread distribution and close association of these species with other mammals, including humans and companion animals, both studies have speculated that they may represent zoonotic sources of HCV infections in humans. Genome sequences and organizational features of NPHV, HCV, GBV-B and rodent/bat hepaciviruses are indeed distinct and therefore the determinants of tropism and pathogenicity may not be functionally interchangeable. Currently, the most widely used HCV animal model is tamarins infected with GBV-B (Simons *et al.*, 1995), a virus of unknown origin but with a demonstrated ability to cause acute hepatitis (Deinhardt *et al.*, 1967). The ability of a hepacivirus to replicate in a broader range of tissues and hosts would therefore provide multiple benefits and opportunities to HCV research.

Despite the existence of clearly homologous regions throughout much of the encoded polyproteins of HCV and NPHV, there are a number of differences that may influence their pathogenicity and replication cycles. In particular, the 3′ and 5′ untranslated regions (UTRs) of NPHV are highly divergent from the corresponding regions of HCV, with the 5′UTR possessing only 66 % nucleotide identity. Although experimental validation has not been conducted, RNA secondary-structure modelling predicts the presence of four RNA stem–loops within the 5′UTR (Burbelo *et al.*, 2012; Kapoor *et al.*, 2011), two of which are similar to the functional domains of the prototypic type IV IRES structure found in HCV (Honda *et al.*, 1996b; Rijnbrand *et al.*, 1995; Tsukiyama-Kohara *et al.*, 1992). However, the NPHV 5′UTR is predicted to contain a large stem–loop at the 5′ terminus that is lacking in HCV (henceforth termed SL1, Fig. 1a). The presence of SL1 is predicted to induce a rearrangement of the immediate downstream region, encompassing microRNA-122 (miR-122)-binding sites. This liver-specific microRNA is essential for HCV replication and RNA stability (García-Sastre & Evans, 2013; Jopling *et al.*, 2005). Finally, the NPHV 5′UTR lacks the domain IV region found within type IV IRES motifs, consisting of a short stem–loop following the pseudoknot. In HCV, this region contributes to polyprotein translation control (Honda *et al.*, 1996a).

**Fig. 1.**
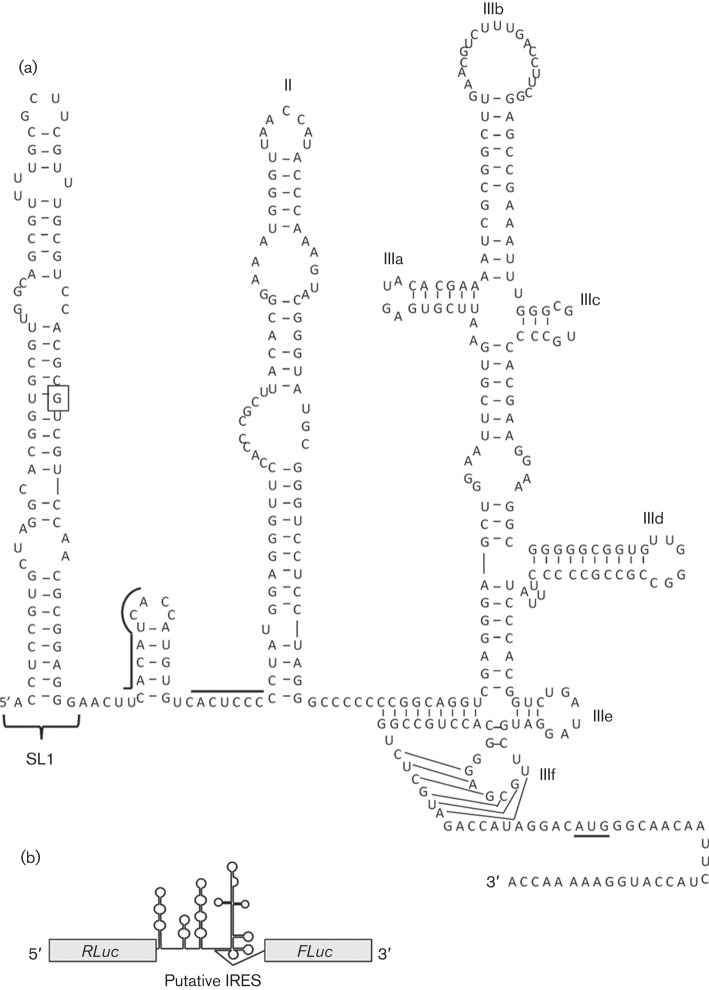
Predicted RNA secondary structures within the NPHV 5′UTR. (a) The nucleotides corresponding to the 5′ terminus and AUG start codons of HCV are boxed and underlined, respectively. Domains II and III(a–f) of the prototype HCV IRES are labelled. Sequences corresponding to microRNA-122 binding sites within HCV are represented by black lines above the primary sequence. Figure adapted from Burbelo *et al.* (2012). (b) Schematic of the bicistronic transcripts used to assess the potential IRES function of hepaciviral 5′UTR sequences.

In common with other RNA viruses, RNA structures play essential roles in both translation and genome replication of flaviviruses. Whether the evident structural differences in the 5′UTRs of HCV and NPHV influence their translation and replication functions was the focus of the current study. NPHV represents an opportunity to dissect the relative contributions of these genomic elements to the replication, pathogenesis and cell tropism of HCV through comparative studies and the development of chimaeric genomes. In this study, we assessed the potential IRES activity of the NPHV 5′UTR, and the contribution of SL1 to this function.

NPHV RNA was isolated from a plasma sample taken from a persistently infected horse (isolate EF369_11J; GenBank accession no. JX948116.1) (Lyons *et al.*, 2012). Two amplicons were produced by reverse transcription-PCR: one encompassing the entire 5′UTR and 30 nt of core coding sequence, and another that excluded the initial stem–loop. A bicistronic vector, in which the *Renilla* (RLuc) and firefly (FLuc) luciferase ORFs are separated by the HCV (gt1b) 5′UTR, was kindly provided by Kensuka Hirasawa (Licursi *et al.*, 2012) (Fig. 1b). This vector encodes 60 nt of the HCV polyprotein ORF prior to the FLuc start codon. An IRES-free control vector (pRZF) was used to assess background FLuc expression. NPHV 5′UTR amplicons were cloned into pRZF; in both cases, 30 nt of coding sequence were included such that the initial 10 residues of the NPHV predicted polyprotein were in frame with that of FLuc. Therefore, both HCV and NPHV bicistronic constructs possessed two Kozak initiation sequences proximal to the FLuc ORF: one encompassing the endogenous AUG of the viral polyprotein and the second being the AUG of FLuc. The relative contribution of each to FLuc expression was not investigated in this study; however, the minimal number of NPHV polyprotein residues (10 aa) would not be predicted to hinder FLuc activity if a chimaeric protein was translated.

To test for NPHV 5′UTR IRES activity, three cell lines were used: Huh-7 (Nakabayashi *et al.*, 1982), Madin–Darby canine kidney (MDCK) (Gaush *et al.*, 1966) and human embryonic kidney HEK293T cells (Graham *et al.*, 1977) were maintained in Dulbecco’s modified Eagle’s medium (DMEM) supplemented with 10 % FBS, 20 mM HEPES, 1 % non-essential amino acids, 100 U penicillin ml^−1^ and 100 µg streptomycin ml^−1^. Both DNA and RNA transfection methods were utilized; for the former, 2 µg plasmid DNA was transfected into 5×10^5^ cells (per well of a six-well plate) using polyethylenimine. Cells were harvested at 48 h post-transfection (p.t.) into passive lysis buffer and a dual luciferase detection assay was performed (Promega). For RNA transfection, uncapped RNA was *in vitro* transcribed from linearized pRZF DNA using a RiboMax T7 kit (Promega), prior to electroporation into 4×10^6^ cells (0.4 cm cuvettes, 950 µF, 250 V). Following electroporation, cells were resuspended in DMEM and seeded into six-well plates (1.2×10^6^ cells per well). Cells were lysed into passive lysis buffer at 6 h post-electroporation (p.e.) and a dual luciferase detection assay was performed.

These results are presented as the ratio of FLuc/RLuc, and indicated that the 5′UTR of NPHV possessed IRES activity in all three cell lines, as the presence of this sequence allowed expression of FLuc at levels significantly elevated over the control pRZF construct, which lacked a putative IRES sequence. This was observed in both DNA (Fig. 2a) and RNA (Fig. 2b) transfection assays. Translation from an NPHV construct lacking SL1 demonstrated that this terminal RNA structure was not required for translation initiation. However, its presence exerted a slight suppressive effect upon IRES activity that was reproducible in all three cell lines during RNA electroporation. Whilst the NPHV IRES was active in three mammalian species, the HCV IRES was inactive in the canine-derived MDCK cells during RNA transfections, suggesting that there might be differences between the HCV and NPHV IRESs in terms of the requirements for cellular IRES *trans*-acting factors. The high levels of FLuc expression observed following DNA transfection of the HCV bicistronic vector in all three cell lines was presumably partially due to ribosomal readthrough of promoter-derived transcripts, and therefore may not be an accurate measure of IRES efficiency when compared with the RNA electroporation method.

**Fig. 2.**
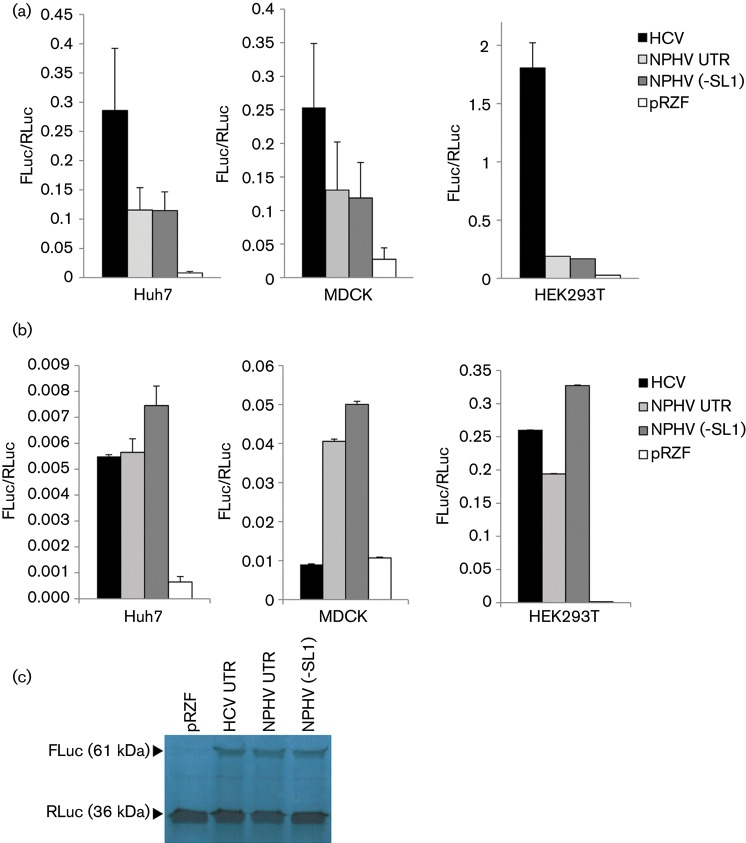
IRES activity of the NPHV 5′UTR. (a) Huh7, MDCK and HEK293T cells were transfected with the indicated DNA plasmids and harvested at 48 h p.t. Cell lysates were assessed for luciferase activity using a dual luciferase kit and the ratio of FLuc to RLuc was calculated (*y*-axis). (b) Cells were electroporated with *in vitro*-transcribed RNA, and harvested at 6 h p.e. for a luciferase assay as in (a). The complete NPHV 5′UTR displayed significantly lower IRES activity than NPHV (−SL1) in all cell lines in this assay (*P<0.05, Student’s paired *t*-test). Data represent the mean (±sem) of three independent experiments. (c) *In vitro* translation products were produced using a rabbit reticulocyte lysate. The presence of both luciferase proteins was detected for all bicistronic vectors, confirming IRES activity.

To further confirm the presence of both luciferase proteins, *in vitro* translation was conducted. Rabbit reticulocyte lysate translation reactions (Ambion) were charged with 1.5 µg RNA transcripts and 4 µCi (148 kBq) l-[^35^S]methionine (PerkinElmer) and incubated at 30 °C for 90 min prior to the addition of 2.5 µl RNase A (1 mg ml^−1^) and incubation for a further 10 min. The reactions were terminated with 2× Laemmli buffer and analysed by SDS-PAGE (12 % acrylamide) and autoradiography. This assay confirmed significant levels of IRES-driven translation of Fluc in all bicistronic constructs, compared with the empty vector pRZF (Fig. 2c).

We next wished to demonstrate the ability of the NPHV 5′UTR to drive cap-independent translation of a hepacivirus subgenomic replicon (SGR), in which the IRES was present at the far 5′ terminus of the transcript instead of being located internally. The HCV IRES within the SGR-Feo(JFH-1) replicon was replaced with either the entire NPHV 5′UTR or a truncated sequence lacking the initial stem–loop [NPHV (−SL1)]. The SGR-Feo(JFH-1) replicon encodes a FLuc–neomycin phosphotransferase fusion protein, driven by the hepacivirus IRES, followed by a downstream encephalomyocarditis virus (EMCV) IRES, which drives expression of HCV NS3-5B (Fig. 3a). The presence of FLuc in cell lysates following electroporation would therefore be indicative of hepaciviral IRES activity. To test whether the NPHV initial stem–loop had an effect on the function of the HCV IRES we also ligated this sequence onto the 5′ terminus of the HCV 5′UTR, to create the SGR-Feo(JFH-1) replicon (SL1-HCV).

**Fig. 3.**
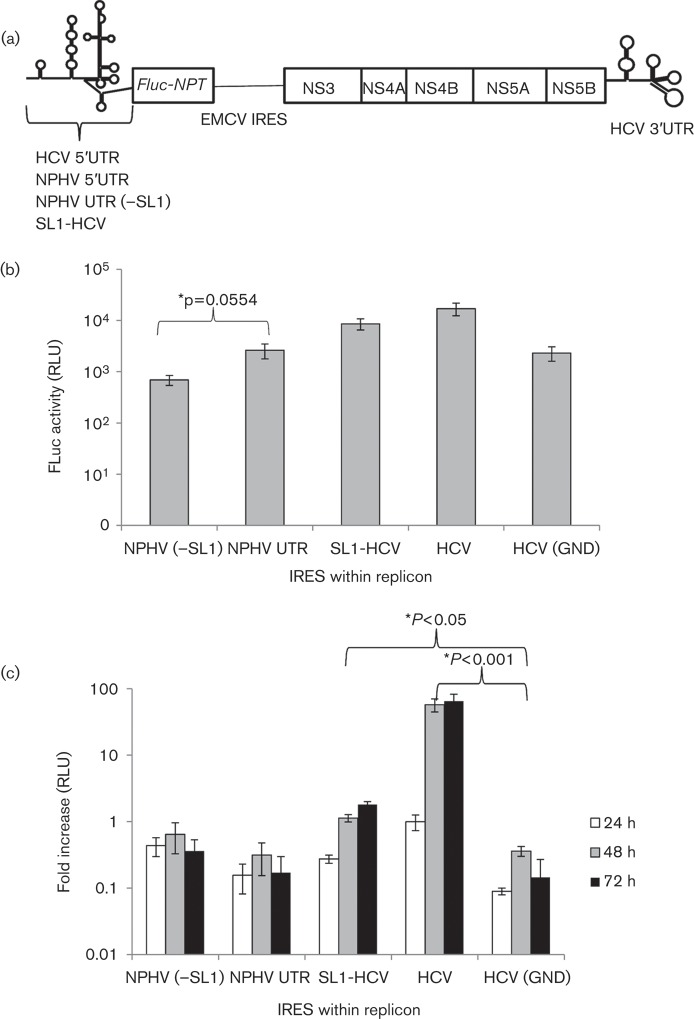
A chimaeric NPHV-HCV 5′UTR impairs replication of an HCV-based SGR. (a) Structure of the SGR: a hepaciviral 5′UTR drives translation of a FLuc–neomycin phosphotransferase (Feo) fusion protein, whilst the EMCV IRES drives translation of HCV NS3–5B. (b) Huh7 cells were electroporated with SGR RNA possessing a range of hepaciviral IRES domains. Lysates were assessed for FLuc activity at 4 h p.e., indicative of initial IRES-driven translation. (c) Huh7 cells were electroporated as described and luciferase activity was measured at 24, 48 and 72 h p.e. The fold increase in luciferase activity compared with the 4 h p.e. value was calculated (*y*-axis) and confirmed replication of the HCV positive-control replicon compared with the replication-deficient HCV (GND) control (*P<0.001, Student’s *t*-test). Both NPHV UTR(±SL1)-containing replicons were replication defective, whilst the presence of the NPHV SL1 impaired HCV replication (*P<0.05, Student’s *t*-test). Data represent the mean (±sem) of three independent experiments.

*In vitro*-transcribed SGR RNA (2 µg) was electroporated into Huh-7 cells as described previously. At 4 h p.e., FLuc activity could be detected from all four constructs and the non-replicating GND control (Fig. 3b), indicating all UTRs possessed IRES function in a replicon system. This result confirmed our previous observation that SL1 is redundant for IRES activity in NPHV. However, the suppressive effect exerted by SL1 in the bicistronic RNA transcript assays was not reproduced in this model; in contrast the NPHV (−SL1) replicon exhibited a slight, although not significant, reduction in IRES activity compared with the entire NPHV UTR (*P* = 0.056).

It has been established previously that the 5′UTRs of hepaciviruses display variation in both their length and predicted structural motifs and these variable structures do not contribute to IRES function. For example, when aligned with HCV, the GBV-B 5′UTR possesses additional sequences at positions 40 and 102 that are predicted to form stem–loops within the region corresponding to domain II of the HCV 5′UTR, whilst still possessing IRES activity (Grace *et al.*, 1999; Rijnbrand *et al.*, 2000). A further difference between hepacivirus 5′UTR sequences is the presence of the domain IV stem–loop in HCV and GBV-B, which is absent in NPHV. We also observed that both NPHV IRES-containing replicons exhibited FLuc activity at least fivefold lower than those containing HCV-based IRES structures (Fig. 3b), despite the insertion of SL1 into one replicon (SL1-HCV). This indicates that the domain IV stem–loop adjacent to the IRES pseudoknot within HCV and GBV-B may influence IRES-mediated translational initiation. Further research is required to assess the significance of the absence of this domain from the NPHV IRES.

Despite the insertions within the 5′UTR, domain I of the GBV-B and HCV 5′UTR is structurally conserved, which is not recapitulated within NPHV. However, this conserved domain is redundant for IRES function in HCV and GBV-B (Luo *et al.*, 2003; Warter *et al.*, 2009), consistent with previous findings that the essential components of a type IV IRES are largely restricted to domains II and III that are conserved across members of the genus *Hepacivirus*. Further support for this hypothesis is provided by studies indicating that the domain III of GBV-B is functionally interchangeable with that of HCV with minimal impact upon replication, which is not the case for domains I and II (Warter *et al.*, 2009).

As the hepaciviral 5′UTR is known to play essential roles in both translation and RNA replication, we assessed the chimaeric SGRs for their ability to replicate in Huh-7 cells. Replicon-RNA-transfected Huh7 cells were maintained for 72 h p.e. Luciferase levels were monitored during this time course and compared with their respective 4 h p.e. values, as this expression level is generally regarded as being a result of initial translation and minimal replication events. Despite displaying initial IRES activity, neither the NPHV 5′UTR-containing replicon nor the NPHV(−SL1) replicon were replication competent, as indicated by a decrease in luciferase activity over 72 h (Fig. 3c). In contrast, the SL1-HCV IRES replicon was replication competent, although this was significantly impaired when compared with the WT SGR-Feo(JFH-1) replicon. It is possible that the presence of SL1 within the SL1-HCV replicon interfered with miR-122 binding, resulting in the impaired replication ability of this replicon. However, this is unlikely to be the sole cause as the NPHV(−SL1) replicon, which would presumably have single-stranded miR-122 sites available for binding, did not replicate, indicating that altered long-range RNA interactions or the lack of a domain IV stem–loop within the pseudoknot might also be contributing factors.

The role of the initial stem–loop of NPHV during replication, and the nature of possible longer term interactions with homologues of the *cis*-acting RNA elements identified in the terminal NS5B coding region of HCV, cannot be determined until an NPHV SGR is available. Future work will concentrate on developing this construct to assess the contribution of SL1 to various stages of the viral replication cycle. The finding that HCV-based replicons possessing a NPHV 5′UTR were unable to replicate indeed suggests that long-range interactions between the 5′- and 3′UTRs are specific to individual hepaciviruses and the mechanism and kinetics of replication are not recapitulated across this genus. This conclusion is further supported by the observation that SGRs possessing chimaeric HCV–GBV-B 5′UTR structures displayed impaired replication kinetics despite retaining their ability to initiate translation (Warter *et al.*, 2009). Even within HCV itself, interactions between the 5′- and 3′UTRs and other *cis*-acting elements affect replication potential (Romero-López *et al.*, 2012). This evidence suggests a scenario wherein the hepaciviruses possess conserved domains involved in IRES function alongside a diverse range of additional structural elements within the far 5′ region of the 5′UTR involved in RNA replication. These domains may contribute to their respective tropisms and pathogenic potential.
